# An insight to rhizosphere bacterial community composition and structure of consecutive winter-initiated sugarcane ratoon crop in Southern China

**DOI:** 10.1186/s12870-022-03463-6

**Published:** 2022-02-19

**Authors:** Abdullah Khan, Hongtao Jiang, Junyao Bu, Muhammad Adnan, Syeda Wajeeha Gillani, Muqing Zhang

**Affiliations:** Guangxi Key Laboratory of Sugarcane Biology, State Key Laboratory for Conservation and Utilization of Subtropical Agro-bioresources, Guangxi University, Nanning, 530004 China

**Keywords:** Sugarcane, Ratoon crop, Rhizosphere, Soil enzymes, Yield

## Abstract

**Background:**

Ratooning in sugarcane is a crucial strategy for ensuring the long-term sustainability of the sugarcane industry. Knowledge gap relating to the interaction between rhizosphere microbiome and ratooning crop, particularly the impact of different sugarcane cultivars on the rhizosphere microbiome in consecutive ratooning, requires additional research. The response of two different sugarcane cultivars, viz ZZ-1 and ZZ-13, were evaluated in consecutive ratooning towards the rhizosphere microbial community and cane morphological characters.

**Results:**

Significant changes in the rhizosphere microbiome were observed in the second ratooning over the years. Several important genera were observed in high abundance during the second ratooning, including *Burkholderia, Sphingomonas, Bradyzhizobium,* and *Acidothermus.* Cultivar ZZ-13 caused more alterations in the rhizosphere microbiome than ZZ-1, resulting in a more favorable rhizosphere environment for sugarcane growth. The genotypes also varied in terms of nutrients and enzyme activity over the years. There were significant differences between the genotypes and year for number of stalks and yield was significant for genotypes, years and genotype × year.

**Conclusion:**

This finding will help to understand thorough interactions between rhizosphere microorganisms and ratoon sugarcane and lay the foundation for promoting and maximizing yield as far as possible. In the future, this work can serve as guidance in sugarcane husbandry, mainly in Guangxi, China.

**Supplementary Information:**

The online version contains supplementary material available at 10.1186/s12870-022-03463-6.

## Introduction

Sugarcane (*Saccharum officinarum* L.), the first significant sugar crop worldwide, is an important cash crop in tropical and subtropical countries. Sugarcane plays a crucial role in farmer economics and provides raw materials to the industry in Southern China [1]. Ratooning is the ability of sugarcane to maintain yield, a desirable character to improve the economics of sugarcane production [2], which is a common practice worldwide and accounts for almost 50% of the total sugarcane planting areas. The sugarcane characteristics associated with ratooning have been studied for possible use in selection criteria while breeding for new varieties [3]. The yield in ratoon crops usually decreased due to the quality degradation with age, disease and pests for a prolonged time [4].

Limited irrigation and the increasing average temperature on the earth surface have affected sucrose accumulation during the ripening and maturation [5, 6]. Ratoon (stubble) crop arises from the buds under the ground from previously harvested crops [5]. Compared to plant crops, ratoon crops typically need less agronomic inputs [7], such as fertilizers, pesticides, etc., to achieve comparable yields. It has been suggested that ratoon crops provided with additional 25–50% nitrogen fertilizers [5] will sustain their yield similar to plant crops. The mortality of tillers and excess nitrogen inputs ultimately affect the dry matter accumulation of ratoon crops, making it necessary to examine the external root environment (rhizosphere).

The rhizosphere, first coined in 1904, was defined as the soil adhering to the surface of roots, which is affected directly by the root system [4]. Apart from water and nutrient uptake, secondary metabolites secreted by the roots, such as carbohydrates and organic acids, provide a favorable environment for the growth and reproduction of rhizosphere microbiota [8]. Therefore, many rhizosphere activities, such as material and energy exchange, plant-soil microbe interactions, are carried in the presence of active soil microbes [9, 10]. Roots provide an optimal location for the microbes to aggregate and interact with plants [11]. Root phenes such as structure might be one factor influencing microbial communities’ assembly, depending on nutrients availability, metabolic pathway, surface area and biotic, and abiotic stresses [12, 13]. Effect of rhizosphere influencing abiotic stress mechanism have been reported in many crops [14, 15]. The microbial community in the rhizosphere differs significantly from that of other soil, such as bulk soil [16]. Various studies revealed that the rhizosphere microbiome is affected by numerous factors, including the microbes’ growth conditions, developmental stages and even continuous cropping [17–19].

Consecutive monoculture is a common practice in sugarcane. Limited studies have been conducted on the selective enrichment of microbial communities through different nitrogen fertilizer rates [20]. Though microbial community structures differ among plant species, informative studies concerning the distinctness of microbial communities in consecutive ratoons are lacking. Therefore, our research was focused on: 1) high throughput sequencing to investigate rhizosphere bacterial communities in consecutive sugarcane ratoon crops, 2) response of rhizosphere microbiome towards consecutive ratooning, and 3) their effect on plant performance and yield.

## Results

### Plant morphological characters and yield of ratooning sugarcane

Analysis of variance revealed significant differences (*P* ≤ 0.05) for the sugarcane morphological traits among genotypes except for plant height and brix % among the years (Table 1). The results showed a 9.12 and 15.69% for ZZ-13 and ZZ-1 stem diameter increase in 2020 compared to 2019, respectively. Stem diameters and sucrose content of ZZ-1 were recorded higher by 15.69 and 3.59% in 2020 over 2019, respectively. Single stalk weight in ZZ-13 and ZZ-1 increased by 9.33 and 25.8% respectively in 2020. While nodes, plant height, internode, the number of stalks, and yield of ZZ-13 were decreased by 4.21, 8.8, 12.3, 27, and 21%, respectively, during 2020 than 2019. Similarly, for the ZZ-1 genotype, plant height, internode length, the number of stalks and yield were reduced by 5.9, 18.1, 15.4, and 22%, while SSW and yield increased by 25.8 and 5% in 2020, respectively, as compared to 2019. In general, our results showed reductions in all traits in 2020 compared to 2019 except stem diameter of both genotypes, nodes, sucrose content and yield of ZZ-1; however, the cane yield was significantly different between the years, genotypes and genotype×year.

**Table 1:** Morphological, sugar content and yield attributes of consecutive ratooning sugarcane

Genotypes	NODES (no)	S D (cm)	P H (cm)	IN L (In)	BRIX (%)	SSW (kg)	No of stalks (ha^**−1**^)	Yield (t ha^**− 1**^)
**2019**								
**ZZ-13**	23.72 ± 1.11b	2.74 ± 0.18b	291.4 ± 9.54a	4.6 ± 0.20a	16.3 ± 0.25a	1.5 ± 0.13a	82,270 ± 7774a	125.32 ± 20.7a
**ZZ-1**	23.7 ± 1.99b	2.23 ± 0.18c	270.6 ± 5.09b	4.4 ± 0.16a	13.8 ± 1.46b	0.93 ± 0.19c	59,900 ± 8480b	56.28 ± 5.46c
**2020**								
**ZZ-13**	22.72 ± 0.63b	2.99 ± 0.15a	263.9 ± 6.80bc	4.03 ± 0.13b	16.2 ± 0.39a	1.64 ± 0.03a	59,959.33 ± 7135b	98.47 ± 7.99b
**ZZ-1**	30.20 ± 1.62a	2.58 ± 0.08b	254.6 ± 8.27c	3.6 ± 0.17c	14.4 ± 0.52b	1.17 ± 0.06b	50,653.33 ± 2800b	59.47 ± 6.06c
**SOV**								
**Genotypes**	18.44 *	56.76*	8.06 ns	4.79 ns	22.46 **	65.81 *	12.60 *	331.8 ***
**Year**	10.03 *	24.12 **	18.00 *	38.22 *	0.05 ns	6.85 ns	12.51 *	17.99 **
**Genotype**×**Year**	18.64 *	0.45 ns	1.58 ns	3.99 ns	0.82 ns	1.02 ns	2.14 ns	25.72 *

Each value is the mean of three replicates with standard error

*S D* Stem diameter, *P H* Plant height, *IN L* Internode length, *SSW* Single stalk weight

^*^P < 0.01

^**^P < 0.001

### Nutrients and enzymes activities of the ratooning cane

**Fig. 1:**
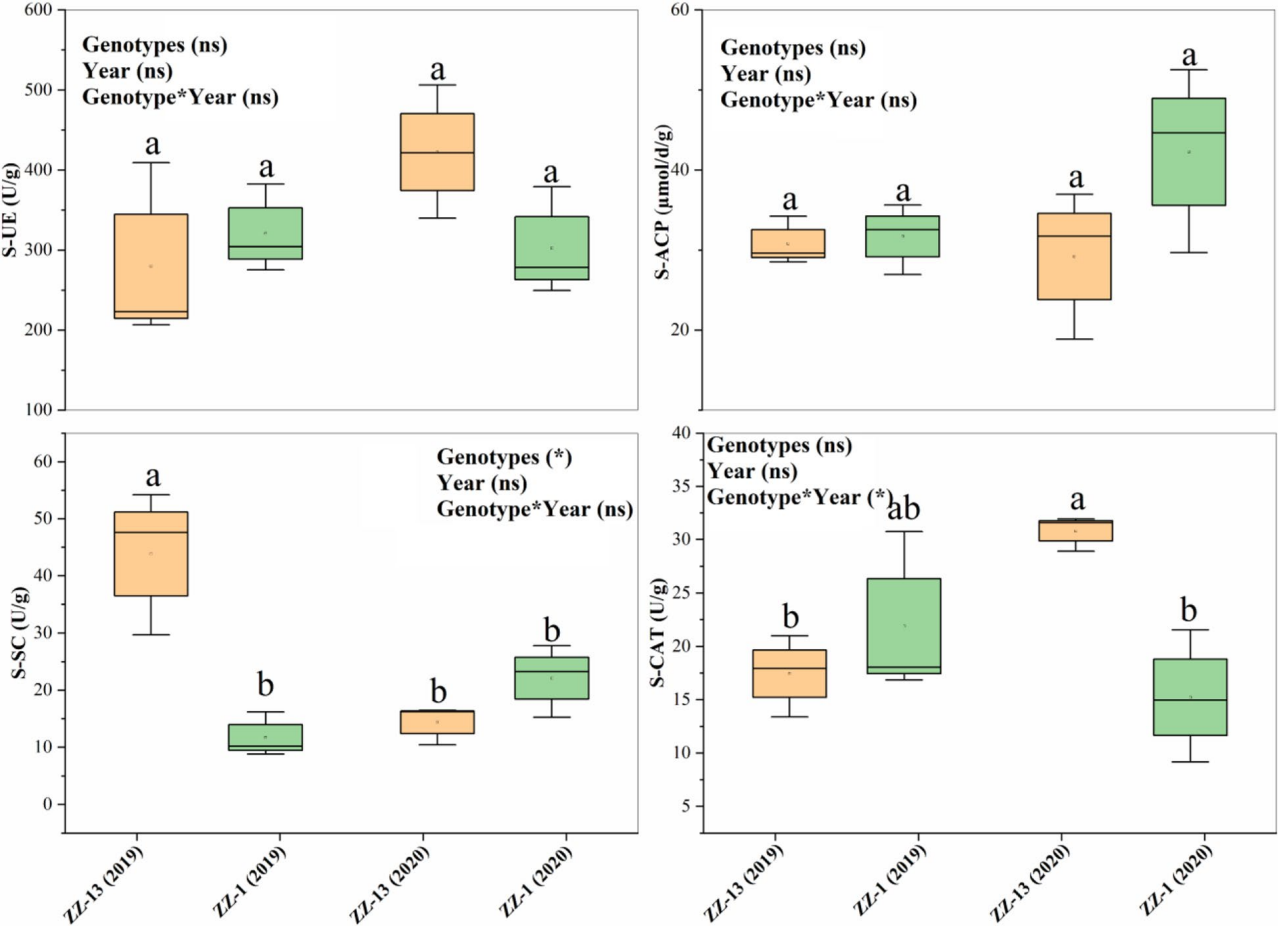
Rhizosphere enzyme activity of consecutive ratoon sugarcane. S-UE, soil urease; S-ACP, soil acid phosphatase; S-SC, soil sucrase; S-CAT, soil catalase. Box plots indicate value for each variety across years. The lowercase letter indicates the differences based on the LSD test (*p* < 0.05). Legends inside each subfigure indicate a significant level at genotypes, year, and genotype*year effect

Soil chemical properties varied significantly (*P* ≤ 0.05) among the 2 years for sugarcane rhizosphere soil, except AK, which was a non-significant effect for year and genotype × year. Compared to 2019, the soil nutrients increased effectively in 2020. These findings suggested that a sugarcane cropping strategy based on ratooning could effectively increase soil fertility. Root morphology across the years was significantly (*p* ≤ 0.05) different. However, their effect was non-significant for genotypes and genotype × year (Table 2). The ZZ-13 cultivar performed better in terms of the number of roots, total root length, and root depth. Enzymatic activity was observed to be increasing with ratooning (Fig. 1). However, their effect was non-significant, except for S-CAT.

**Table 2:** Effect of consecutive ratooning sugarcane on root morphology and soil nutrients

Genotypes	Roots (no)	TRL (m)	Depth (m)	SOC (g/kg)	AN (mg/kg)	AK (mg/kg)	AP (mg/kg)
**2019**							
**ZZ-13**	66.6 ± 15.1a	144 ± 28.5a	0.20 ± 0.03b	21.5 ± 1.6b	59.1 ± 2.8c	209.2 ± 3.5b	31.1 ± 2.5b
**ZZ-1**	52.4 ± 9.29a	142 ± 49.1a	0.26 ± 0.08a	34.2 ± 1.4a	101.2 ± 3.9a	193.4 ± 4.2bc	42.6 ± 3.21a
**2020**							
**ZZ-13**	38.5 ± 8.2b	65.3 ± 18.25b	0.13 ± 0.03c	24.96 ± 1.5b	75.3 ± 2.5b	235.1 ± 3.5a	45.2 ± 4.5a
**ZZ-1**	28.8 ± 7.8b	75.14 ± 16.4b	0.12 ± 0.02c	33.9 ± 1.4a	113.4 ± 2.19a	211.3 ± 4.2b	49.5 ± 6.6a
**SOV**							
**Genotypes**	1.5 ns	0.03 ns	4.54 ns	2260.2 **	1702.7 **	7007.1*	216.6 *
**Year**	29.5 *	21.1*	21.74 *	34.7 *	2.57 ns	2.38 ns	93.5 *
**Genotype×Year**	1.18 ns	0.08 ns	0.80 ns	76.95 *	457.4 *	928.4 ns	63.93 *

Each value is the mean of three replicates with standard error

*TRL* Total root length, *SOC* Soil organic matter, *AN* Total nitrogen, *AK* Available potassium, *AP* available phosphorous

^*^P < 0.01

^**^P < 0.001

### Correlation analysis among different morphological, enzymes, and nutrients

**Fig. 2:**
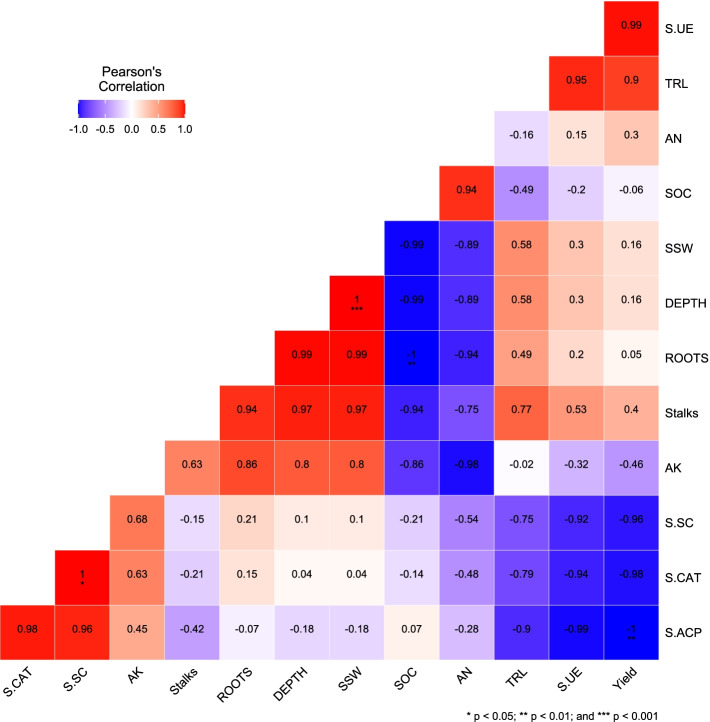
Pearson correlation analysis for different parameters among consecutive ratooning. A color gradient denoting Pearson correlation coefficient displayed pairwise comparisons of environmental factors and morphological characters

The yield was strongly positively correlated with TRL (0.90), S-UE (0.99), and the number of stalks (0.40), and moderately correlated with SSW (0.16), depth (0.16). Number of stalks was positively correlated with SSW (0.97), AK (0.63), S-UE (0.53), depth (0.97) TRL (0.77), and number of roots (0.94) (Fig. 2).

### Microbial diversity analysis and sequencing details associated with consecutive ratooning

**Fig. 3:**
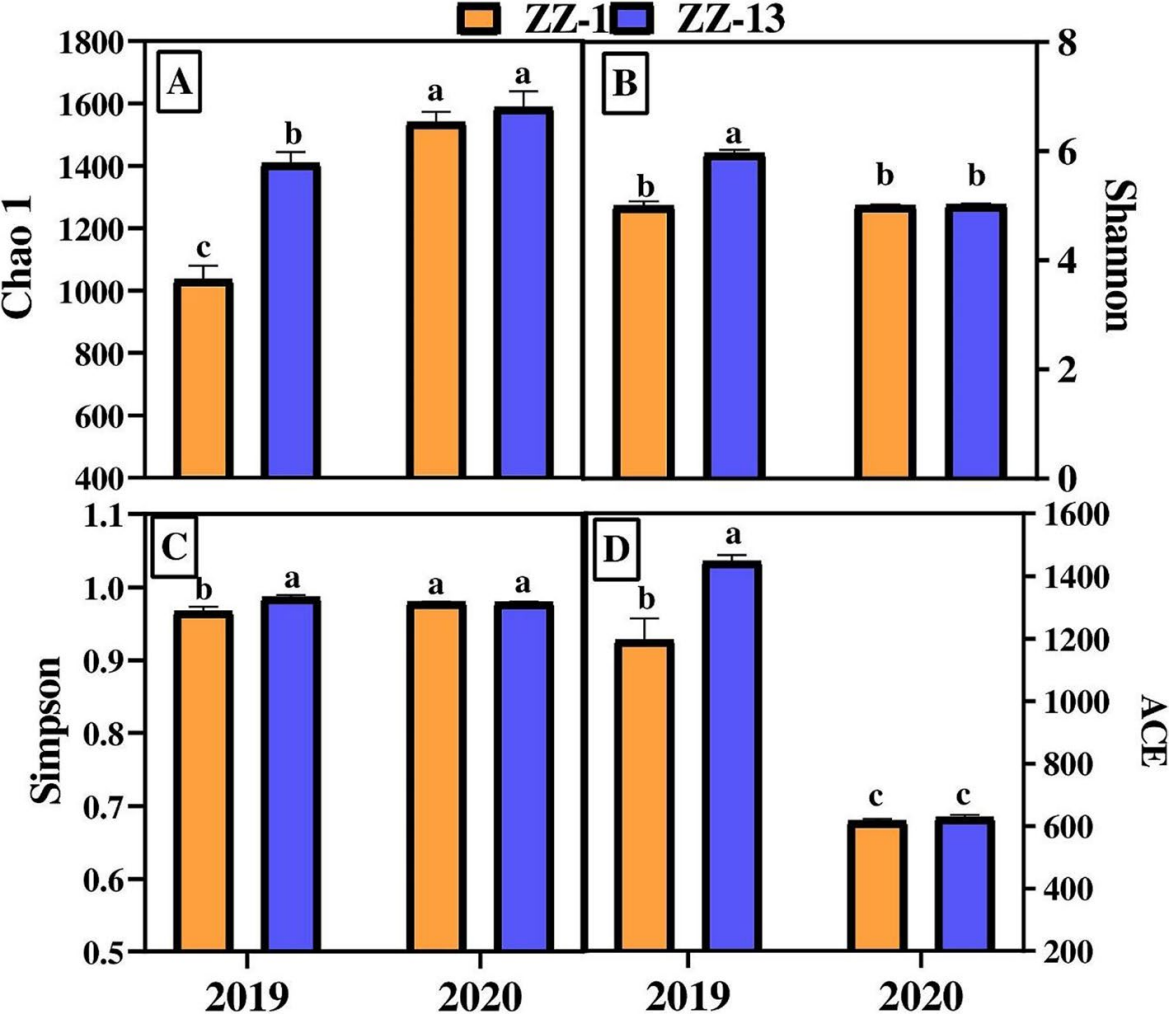
Alpha diversity indices for consecutive ratooning sugarcane. Different lowercase letters indicate the difference based on the LSD test (*p* ≤ 0.05). The legend on the top indicated the varieties. **A-D** chao1, Shannon, Simpson, and Ace index mean values from replicated samples for each variety across 2 years

A total of 323,990 and 267,694 valid reads were obtained from the consecutive ratooning sugarcane rhizosphere in 2019 and 2020, respectively. The details are given in supplementary file S1. A total of 8988 and 4310 OTUs were detected at 97% similarity in the first and second ratoon consecutively. The rhizosphere bacterial community richness and diversity were calculated using Chao1, Ace, and Simpson, respectively. There were significant differences for OTU richness estimated by Chao1 (*p* ≤ 0.05) and bacterial diversity estimated by Shannon index (*p* ≤ 0.05) across consecutive years for the tested genotypes (Fig. 3). ZZ-13 showed a higher bacterial diversity and OTU richness in both years compared to ZZ-1. ZZ-13 exhibited more species richness in 2019 and 2020 as compared to ZZ-1. The Simpson index was high for ZZ-13 (0.9863) in 2019 than ZZ-1 (0.9669); however, in 2020, there were no significant differences between the two genotypes for Simpson index. The richness and evenness of bacterial community species varied across the two sugarcane crop cycles, indicated that the changes in soil chemical properties and root growth/exudates had modulated the microorganisms in the rhizosphere selectively.

### Microbial community composition in consecutive ratooning

**Fig. 4:**
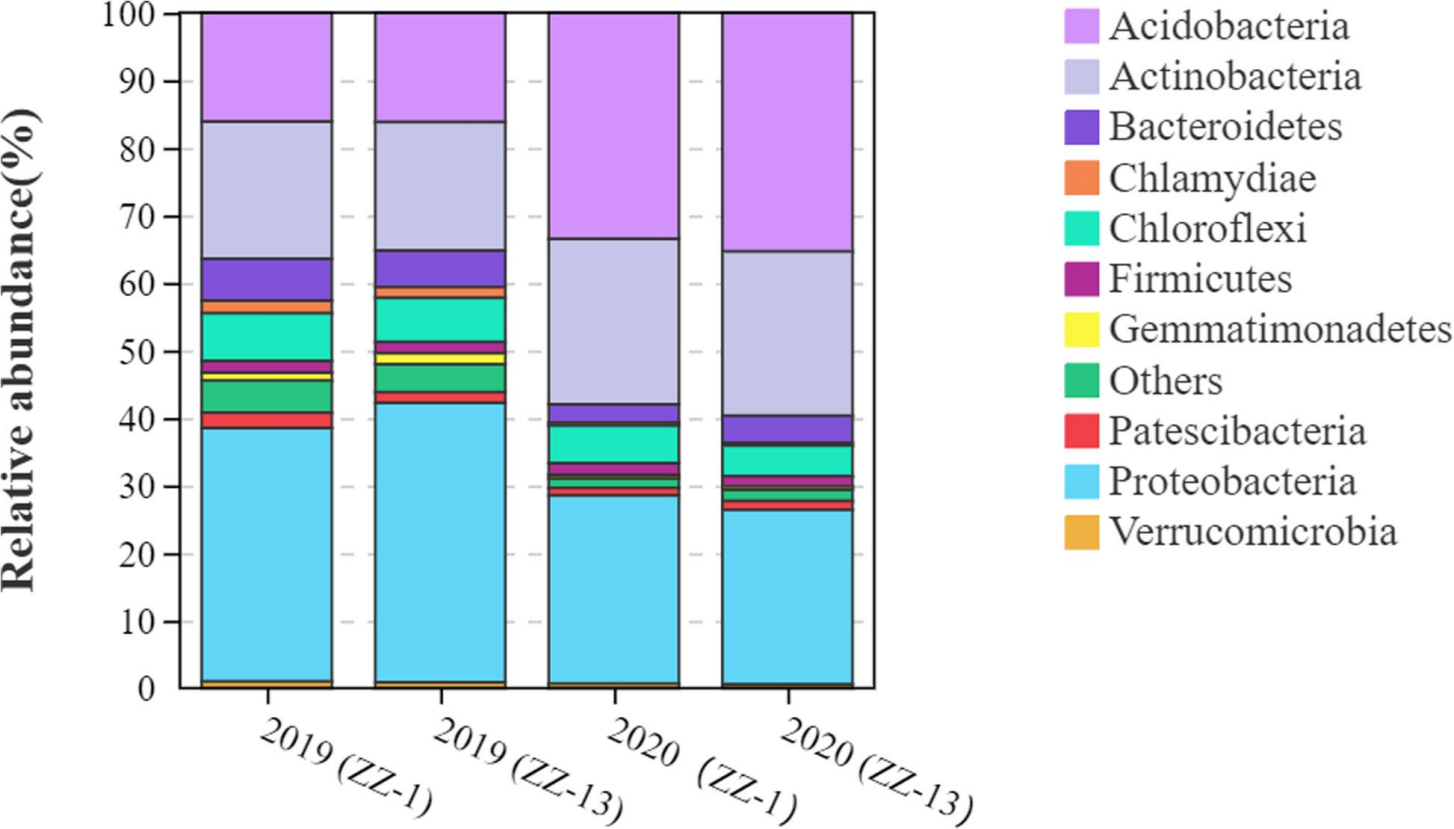
Relative abundance of major bacterial phyla in the rhizosphere of ZZ-1 and ZZ-13 in consecutive ratooning sugarcane

**Fig. 5:**
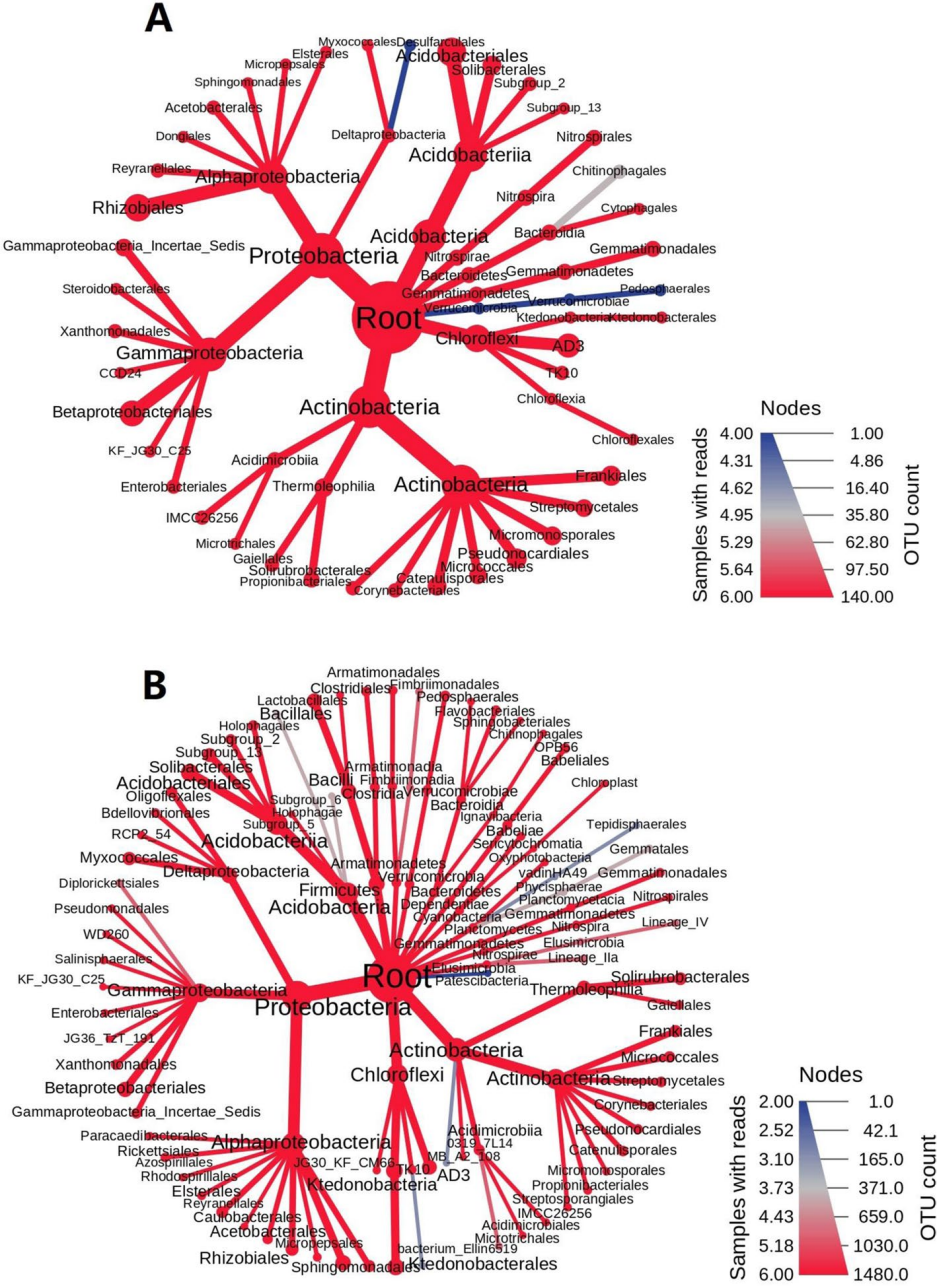
**A** taxonomic abundance of rhizosphere samples in 2019; **B** Taxonomic abundance of rhizosphere samples in 2020. Heat tree analysis illustrating the taxonomic differences between the 2 years. The color gradient and the size of the node, edge, and label are based on samples and their OTU count

Based on the top 10 most abundant bacterium phyla analysis, sugarcane rhizosphere dominated proteobacteria, actinobacteria, acidobacteria, chloroflexi, bacteriodetes, and firmicutes (Fig. 4). Proteobacteria accounted for almost 47% of the total number of species present in the rhizosphere of both ratoon crops. However, the proportion of some bacterial phyla varied during the second ratooning. For example, an abundance of acidobacteria, chloroflexi, and firmicutes increased in the second ratooning. The results suggested the ability and response of bacterial communities towards ratooning crops. The abundance and occurrence of major bacterial taxa with increasing OTU number were displayed in both years (Fig. 5).

### Microbial community structure

**Fig. 6:**
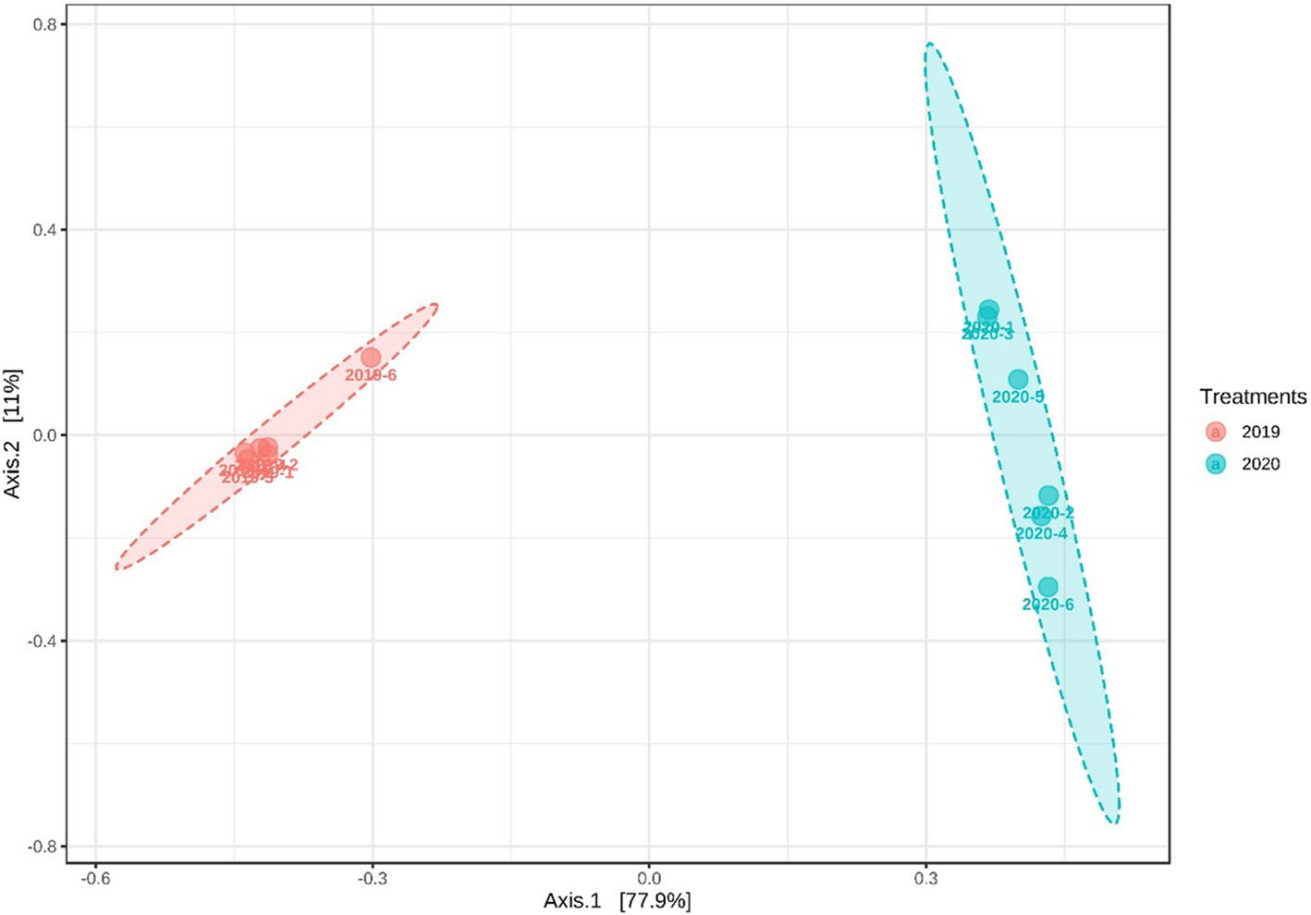
Beta diversity analysis. Principal coordinates analysis of the rhizosphere soil among 2 years using the Bray-Curtis algorithm. Different colors and ellipses indicate the year

**Fig. 7:**
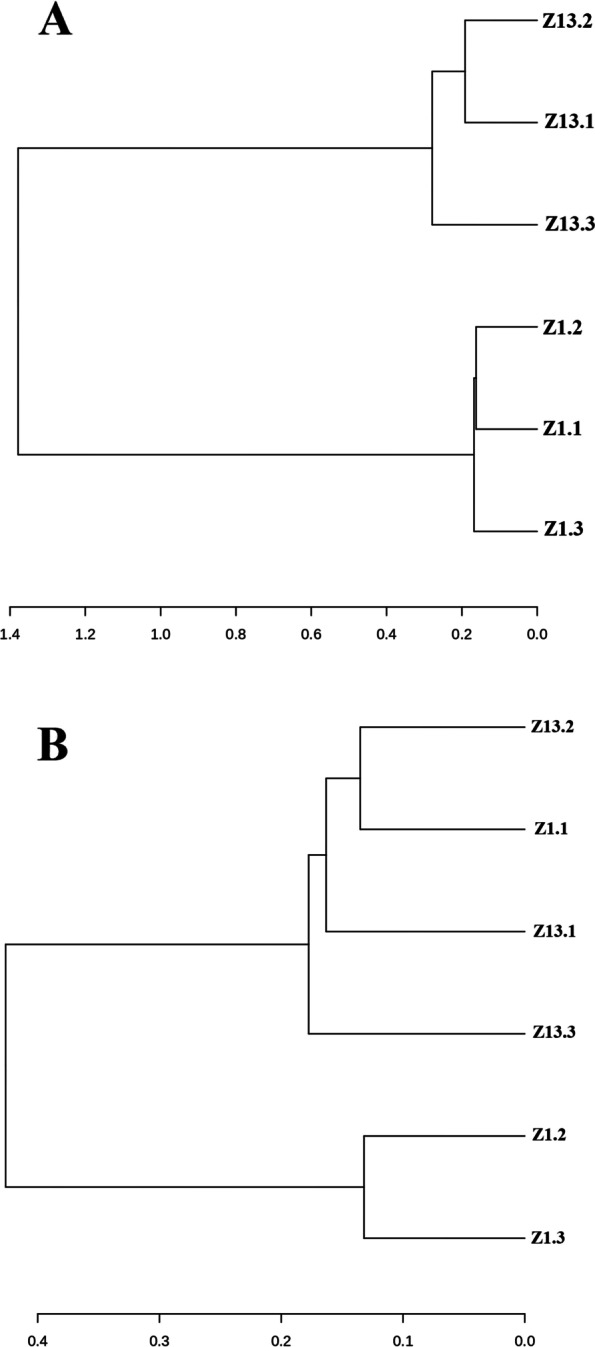
Hierarchal cluster analysis. **A** cluster analysis of rhizosphere samples during 2019 in the studied genotypes; **B** cluster analysis of rhizosphere samples during 2020

Principal coordinate analysis (PCA) using the Bray-Curtis algorithm was carried out to investigate differences among the rhizosphere community across ratooning. The microbial community across the years was different from each other (Fig. 6). The two-year samples were separated from each other, indicating different microbial compositions among the years. The first two principal components explained 88.9% of the total variation in the bacterial communities. The PC1 and PC2 explained 77.9 and 11% variation, respectively. Moreover, similar results were also observed in hierarchical cluster analysis of the two varieties across ratooning years (Fig. 7). According to the results, the rhizosphere soil samples in 2019 formed two groups in which ZZ-1 and ZZ-13 clustered separately, while in 2020, the samples clustered into three groups.

## Discussion

The rhizosphere microbiome has rapidly evolved and has been a focus of interest for research communities in the last few decades. Plant efficiently enhanced soil microbes among soil and roots. The release of plant roots intrigues soil microorganisms nearby. Variations in microbial community composition do exist, however, between plant species and even between genotypes [21]. In this study, the differences between the plant morphological characters rhizosphere bacterial populations of consecutive ratooning sugarcane were investigated using Illumina MiSeq high-throughput sequencing.

The diversity and abundance of bacterial populations were higher in ZZ-13 than ZZ-1, implying that the link between bacteria and the soil environment was more complicated, and the ecosystem was more stable in ZZ-13. High soil microbial diversity is beneficial for positive plant-soil feedback and N nutrition availability in soil [22]. Microbial diversity and abundance may differ amongst cultivars of the same species due to biomass and root shape. For example, high-yielding cultivars have significantly increased the root porosity and an abundance of certain microorganisms inferring that their large roots and porous soil eases gas exchange in the rhizosphere and influence the rhizosphere bacterial community [23]. It can also be observed from the field agronomic data of the two cultivars (Table 1), which showed that ZZ-13 was substantially better performing than ZZ-1. Root morphology might have also affected the bacterial communities. Fine roots might convey more nutrients and metabolites with a considerably large surface area from more extensive roots [24]. These characteristics could make them ideal for microbial colony proliferation, suggesting that plants with more extensive roots might form beneficial associations with a broad microbial community [25, 26], which appeared to be mirrored in ZZ-13. The beta diversity analysis revealed that the rhizosphere microbial communities were completely different during the consecutive ratooning (Fig. 7). This indicated that the relative abundance of bacteria in the sugarcane rhizosphere could be explained by their quantity and composition of root exudates [27]. The majority of these secretions are primary metabolites like carbohydrates, organic acids, and amino acids [28], which are secreted by plant roots and spread to the soil [29], affecting the content of nutrients like C and N in the rhizosphere soil [30], and thus enriching the microbial community. The root exudates from sugarcane with different genotypes had a different effect on microbial communities’ colonization, indicating that sugarcane root exudate could affect bacterial community shaping in the rhizosphere. Hence many root parameters such as total root length and diameter might also improve the absorption rate of N [31, 32]. From the analysis, consecutive ratooning and different cultivars substantially influenced the dominant bacterium species.

In comparison, Chloroflexi was much more abundant in ZZ-13 compared to ZZ-1 in the 2nd ratooning. Many bacterial species belonging to Chloroflexi are involved in nitrite oxidation [33], while those of alpha proteobacteria have been shown to have N fixing abilities [34]. Generally, because of changes in the root exudates and other metabolites, root morphology ZZ-13 and ZZ-1 might produce diverse rhizosphere bacterial populations, resulting in the variation in the use of nutrients, including N and P and the formation of different network topologies.

The differences between the bacterial communities in the consecutive ratooning rhizosphere were most perceptible in Actinobacteria, Acidobacteria, Chloroflexi, and firmicutes. The proportion of Actinobacteria and Acidobacteria increased in the 2nd ratooning by 7 and 52.8%, respectively. Acidobacteria predominated in the 2nd ratoon crop, while proteobacteria, a vital plant growth promoter was observed in the 1st ratoon rhizosphere. This was attributed to the improved crop growth and yield, as evidenced by morphological data. However, the relative abundance of Proteobacteria decreased with the ratooning of sugarcane, which needs further study to clarify that whether proteobacteria communities play a similar role in ratooning. The diversity of the soil microbial population was reflected in the abundance of soil microorganisms. The leading genera found in this study varied substantially between consecutive ratooning. Sugarcane engaged in complicated interactions with rhizosphere bacteria throughout the ratooning process [5], as evidenced by the distribution of dominating bacteria in the rhizosphere soil. Microbial diversity in the rhizosphere is vital for plant growth and health [35]. The dominating taxa in the sugarcane rhizosphere were *Conexibacter, Acidothermus, Sphingomonas, Burkholderia,* and *Bradyzhizobium* (Supplementary File S2). In previous studies, *Sphingomonas* has been found to potentially break down environmental contaminants and stimulate plant absorption and growth [36]. *Sphingomonas* has also been the predominant antimicrobial agent in soil communities and limits plant pathogenic fungi growth [37]. *Bradyrhizobium* is a widespread soil bacterium capable of forming symbiotic connections with plant roots and nitrogen fixation [38]. However, the impacts of *Sphingomonas, Bradyzhizobium,* and *Burkholderia* on sugarcane ratooning ability, nutrients uptake, and root growth in the rhizosphere of sugarcane need to be investigated further. Compared with the first sugarcane ratoon, some beneficial microbial groups such as *Burkholderia, Bacillus, Occallatibacter,* and *Bradyzhizobium* increased significantly in the consecutive sugarcane ratoon crop. Such beneficial microbes might play a vital role in the ratooning sugarcane crop.

The assessment of soil environmental parameters revealed that SOC, AN, AK, and AP were significantly different between ZZ-13 and ZZ-1 (Table 2). A slight increase in the nutrients was observed, which might be attributed to many factors, including the degradation of leftover leaves, stem cuttings, and stubbles after harvesting. The microbial communities give accessible N to plants via biological N fixation and organic form mineralization, limiting N loss by retaining it in humus. Plant-microbial interactions impacting P usage are poorly understood. However, Arabidopsis mutants might increase bacterial communities in phosphate-rich soil, compete for phosphate with plants, and promote phosphate fixation [39]. Plant roots could assemble particular bacteria, allowing the P cycle to be enhanced [40].

The relationship is influenced by the rhizosphere’s bacterial community and the soil particles’ enzyme activity [41]. Soil enzymes are bioactive proteins found in soil, which are mostly produced by bacteria [42]. Soil enzymes play a crucial role in the soil nutrient cycle. They are intimately linked to soil fertility and ecological sustainability, and their increased activity can boost nutrients input capability from plant soils [43]. Our research discovered that S-UE, S-ACP, and S-CAT activity increased with consecutive ratooning most noticeably in ZZ-13 rhizosphere soil, except S-SC. Urease is involved in the soil N cycle, reflecting soil health and vitality [44]; urease activity rises with the increased SOC in soil and has been linked to P and soil catalase (S-CAT). However, the physiological stimulus of root growth, such as branching or root hair development, mediated by Indole-acetic-acid and other hormones, could also increase plant P uptake. Furthermore, alterations in the microbial structure in the rhizosphere might impact the activity of numerous enzymes in the soil and affecting soil nutrients indirectly.

## Conclusion

We compared the differences in the rhizosphere microbiome of two sugarcane genotypes, namely ZZ-13 and ZZ-1, in consecutive winter-initiated ratooning. Based on morphological field data and rhizosphere soil characteristics, including enzymes, nutrients, and bacterial microbiome, ZZ-13 was a promising genotype suited for ratooning in Guangxi, China. Furthermore, the abundance of Actinobacteria and Acidobacteria in follow-up ratoon suggested specific feedback towards consecutive ratooning. Alpha diversity metrics further confirmed more species richness and diverse bacterial communities. This finding will help understand thorough interactions between rhizosphere microorganisms and ratoon sugarcane and lay the foundation for promoting and maximizing yield as far as possible. Currently, we are identifying the response of ratoon sugarcane rhizosphere bacterial communities towards different levels of chemical fertilizers. Results will most like to have a substantial effect in enhancing sugarcane crop husbandry.

## Methods

### Plant material and experimental site

The sugarcane varieties used in this experiment were ZZ-1 and ZZ-13. ZZ-1 is offspring of Chinese germplasm viz. ROC25 × YZ89–7, while ZZ-13 is offspring of foreign germplasm viz. HOCP01–157 × CP14–0969. The foreign germplasm from USA along with Chinese germplasm was bred in state key laboratory for conservation & utilization of subtropical agro-bioresources and cultivated in field station of the Guangxi University in Fusui, Chongzou, China. Both the varieties are cultivated species used for sugar production in the Fusui region. The experimental research on these cultivars were compiled with the national guidelines of China.

**Fig. 8:**
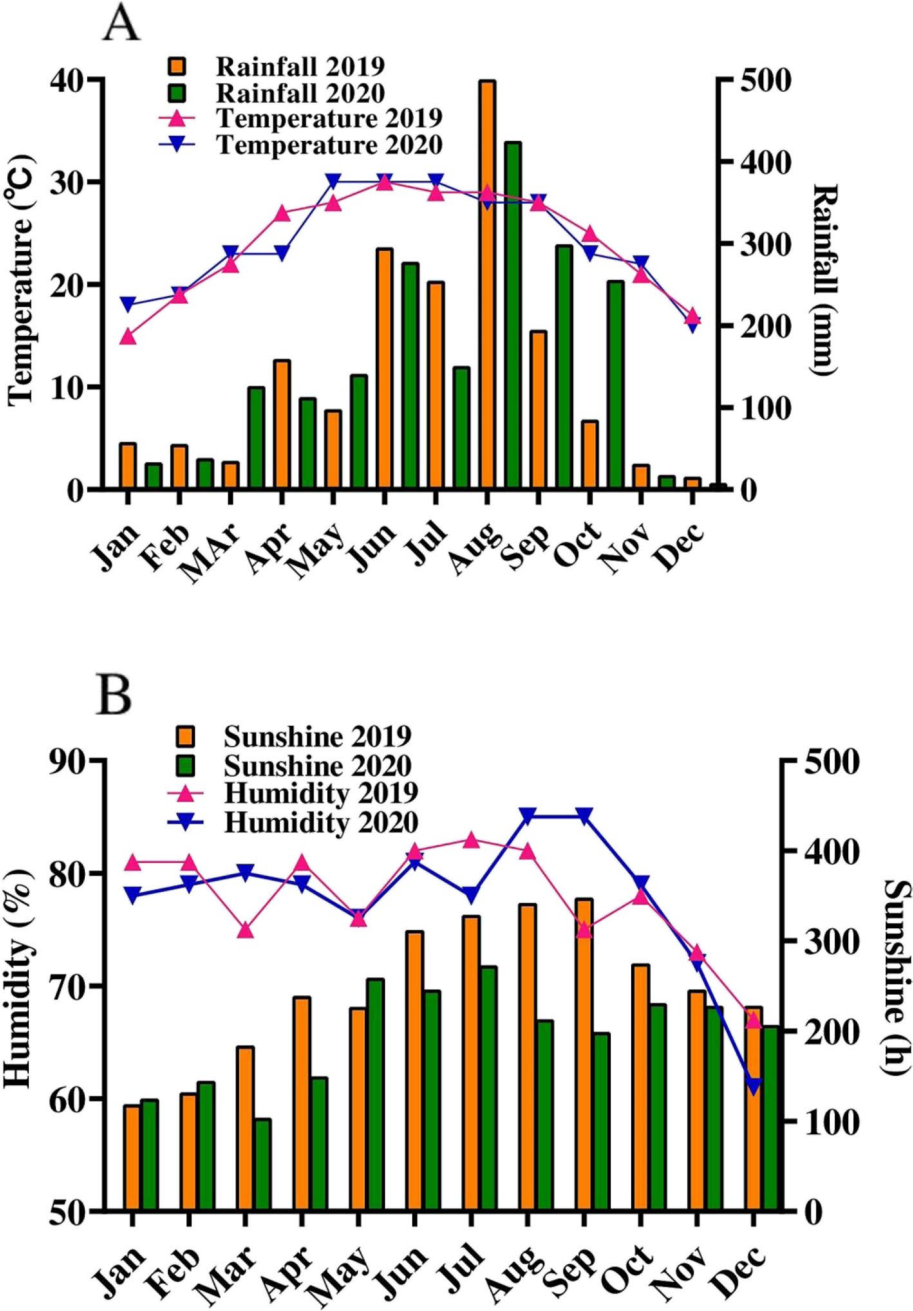
**A** Monthly mean rainfall and temperature data during the 2 years, **B** Humidity and sunshine data during the 2 years

The experiment was carried out in the field station of the Guangxi University in Fusui, Chongzou, China (22^0^38′06″N,107^0^54′15″E). The station is one of the major sugar planting areas in Guangxi Zhuang Autonomous Region. The climatic conditions for the region over the 2 years are given in Fig. 8. The annual mean temperature during both years was 24.1 °C, while the total annual precipitation during 2019 and 2020 was 1770 and 1872 mm respectively. The annual mean humidity of the region during 2019 and 2020 was 77.83 and 77.75% respectively. The region annual average sunshine was 247.41 h in 2019 and 197.33 h during 2020.

### Experimental design and samples collection

The experiment was arranged in a randomized complete block design having three replicates per genotype. The total subplot area was 120 m^2^. Each variety was grown in a 30 m long block with 2 m line to line distance and 30 cm plant to plant distance. Agronomic practices such as weeding, fertilization etc. were kept uniform for all the replicates. During both growing seasons, the crop was utterly reliant on natural precipitation.

Field data was recorded over two consecutive years (2019–2020) in late December when the sugarcane was in the ripening phase. Morphological data, including the number of nodes, plant height, stem diameter, and internode length, were recorded on 30 plants per replicate, and their average value was computed. Each soil was analyzed in three replicates. The plant height was calculated with the help of a meter rod. The number of nodes in each plant was counted, and then their average value was taken. Stem diameter and internode length were calculated with the help of a vernier caliper on every 10th internode from the plant top, and the average value was recorded. The soil adhered to the roots was collected as rhizosphere soil and was divided into three parts for further analysis. The 1st part was kept in a 50 mL sterilized centrifuge tube and stored at − 80 °C for DNA extraction, the 2nd part was kept for nutrient determination, and the 3rd part of the soil was used for enzyme measurement. Each step was performed with three biological replicates.

### Estimation of sucrose and theoretical yield

Before harvesting, sucrose content was measured using a portable refractometer ATAGO Pocket PAL-1 (Atago Co. Ltd., Tokyo, Japan). The theoretical cane production was calculated according to the following equation [45].

- Single stalk weight (kg) = (stalk diameter (cm))^2^ × (stalk height (cm) – 30) × 1 (g/cm^3^) × 0.7854/1000.
- Cane yield (t/ha^− 1^) = single stalk weight (kg) × stalk numbers (no ha^− 1^) /1000.

### Root analysis and soil chemical properties

Root samples were washed with tap water to remove the soil particles using a 1 mm sieve mesh to minimize root loss. The root morphology was then calculated by scanning the root samples with an STD4800 WinRhizo Scanner (Regent Instruments Inc., Canada). The sieved soil samples using 2 mm mesh were subjected to different experiments to calculate SOC, AN, AP, and AK. Soil samples were oxidized with K_2_Cr_2_O_7_.H_2_SO_4_ and titrated with FeSO_4_ to determine the SOC content [46]. The soil AN was determined according to the procedure defined by [47]. Available phosphorous was calculated by the method prescribed by [48], and AK quantity was measured following the method of [49]. Similarly, acid phosphatase (S-ACP), catalase (S-CAT), urease (S-UE), and sucrase (S-SC) activity were measured using soil enzyme kits [50].

### DNA extraction and PCR amplification

According to the manufacturer’s instructions, rhizosphere soil DNA was extracted from each sample using the FastDNA spin kit for soil. The quantity of extracted DNA was measured by NanoDrop 2000 (Thermo Fisher Scientific, Wilmington, USA). The bacterial v5-v6 region of 16 s rRNA was amplified by 799F (forward primer, 5-AACMGGATTAGATACCCKG-3) AND 1193R (reverse primer 5-ACGTCATCCCCACCTTCC-3) [51]. The DNA samples were used as the template for amplification. In 25 μL of the reaction mixture, the PCR reactions were carried out containing 20 ng DNA template, 0.5 μL dNTP, 10 μL Buffer, 0.25 μL DNA polymerase, 5 μL of High GC enhancer, and 1.0 μL of each primer. The PCR thermal conditions were as follows: The initial denaturation was carried at 98 °C for 5 mins followed by 25 cycles at 94 °C, 52 °C for 30 s (annealing), 72 °C for 30 s (extension), and 72 °C for 10 mins (final elongation). PCR amplifications were performed using a BioRad S1000 thermocycler (Bio-Rad Laboratories, CA, USA). The products were mixed equally after the reaction, and the target bands were detected using 2% agarose gel electrophoresis. The targeted bands were recovered through QIAamp DNA Micro Kit (Qiagen, Valencia, CA, USA). Subsequently, DNA libraries were constructed with the Illumina TruSeq DNA sample preparation kit (Illumina, San Diego, CA, USA). High throughput sequencing of 16 S rRNA was carried out using an Illumina HiSeq2500 platform, and 250 bp paired-end reads were generated (Gene Denovo  Biotechnology Co., Ltd., Guangzhou, China).

### Data analysis

Raw tag sequences were screened for quality and assembled to clean reads using FLASH software. The clean reads identified were assigned to the corresponding sample to obtain valid sequences for each sample. QIIME (Quantitative Insights Into Microbial Ecology v.1.9.0) software was used to further carry out the downstream analysis. The operational taxonomic unit (OTU) was assigned to representative sequences by processing the pair end data as an input file in QIIME software. Using the UCLUST algorithm and Green genes as a reference database, OTUs were picked up at a 97% similarity threshold [52]. Each OTU sequence from both ratoon samples represented the taxonomy, including phylum class, order, family, genus, and species within each sample. Microbiome Analyst [53] was used to further analyses the output OTU table. The input data were rarefied to the minimum library size with total sum normalization using the default functions. The sequences were filtered at a minimum of 4 with a 20% prevalence in the sample, and the low variance filter was set at 10% using the interquartile range. Relative abundance of different taxa was calculated for each sample. The diversity indices including Chao1, ACE, Simpson, and Shannon were calculated for each sample, and a rarefaction curve was drawn using Mothur (v.121.1). Within a single sample, the diversity was described by alpha diversity. Beta diversity analysis was carried out with the R function to evaluate differences or similarities between the consecutive ratooning.

### Statistical analysis

Mean values obtained from each replicate were subjected to test the significance and effect of consecutive ratooning on the studied parameters. Two-way analysis of variance (ANOVA) was used to test the differences between genotypes among years, then the individual means of each parameter was compared by LSD test. Origin software was used to illustrate figures.

## Supplementary Information

**Additional file 1: Fig. S1.** Number of reads in rhizopshere soil of each sample during the 2 years. **Fig. S2.** The abundance of dominant genera in rhizosphere soil of each sample during the 2 years.

## Data Availability

Additional data is available on reasonable request from the corresponding author.

## Abbreviations

AK: Available potassium
AN: Available nitrogen
AP: Available phosphorous
INL: Internode length
PH: Plant height
S-ACP: Soil acid phosphatase
S-CAT: Soil catalase
SD: Stem diameter
SOC: Soil organic carbon
S-SC: Soil sucrase
SSW: Single stalk weight
S-UE: Soil urease
